# A retrospective study on post-traumatic stress disorder in fathers of preterm infants in the NICU and the effectiveness of kangaroo care intervention

**DOI:** 10.3389/fpsyt.2026.1670036

**Published:** 2026-06-04

**Authors:** Changying Zhu, Zhenzhen Li, Jianmin Zheng, Dongxue Chen, Ping Chen, Jianlan Chen

**Affiliations:** 1 Neonatology, The First Hospital of Putian, Putian, Fujian, China; 2 School of Nursing, Putian University, Putian, Fujian, China; 3 Xiamen Xianyue Hospital(Department of Nursing), Xianyue Hospital Affiliated with Xiamen Medical College, Fujian Psychiatric Center, Fujian Clinical Research Center for Mental Disorders, Xiamen, Fujian, China

**Keywords:** a retrospective study, kangaroo care, neonatal intensive care unit, post-traumatic stress disorder in fathers, preterm infants

## Abstract

**Objective:**

The aim of this study was to investigate the factors associated with the occurrence of screen-positive post-traumatic stress disorder (PTSD) symptoms in fathers of preterm infants in the neonatal intensive care unit (NICU) and the effects of the application of kangaroo care measures.

**Methods:**

This study comprised two components: a retrospective cross-sectional analysis to identify factors associated with PTSD in fathers of NICU preterm infants, and a prospective, quasi-experimental pre–post intervention study to evaluate the effects of kangaroo care. For the cross-sectional part, 251 fathers were included and grouped based on the Chinese version of the Perinatal Posttraumatic Stress Disorder Questionnaire (PPQ-C) scores (screen-positive group, *n* = 62; screen-negative group, *n* = 189). Univariate and multivariate logistic regression analyses were performed to identify independent risk factors, and a nomogram prediction model was constructed and internally validated using receiver operating characteristic (ROC) curves. For the intervention part, kangaroo care was implemented, and its effects were analyzed by comparing PTSD symptom rates, illness uncertainty, social support, and depressive symptom scores before and after the intervention.

**Results:**

Significant differences were found in illness uncertainty, social support, depressed mood, infant birth weight, paternal occupation, and education level (*p* < 0.05). Logistic regression confirmed these as independent risk factors [odds ratio (OR) = 1.053, 0.893, 1.300, 2.564, 2.939, 2.389, *p* < 0.05]. The prediction model [area under the curve (AUC) = 0.872, 95% confidence interval (CI): 0.817–0.927] showed high accuracy. After kangaroo care, the rate of screen-positive PTSD symptoms, illness uncertainty, social support, and depressed mood improved significantly (*p* < 0.05).

**Conclusion:**

The risk of screening positive for PTSD symptoms and uncertainty of illness, social support, depressive symptoms, child birth weight, occupation, and education level are all independent risk factors. Screening high-risk groups using a nomogram prediction model and implementing kangaroo care interventions were associated with improvements in these psychological outcomes. These findings provide a preliminary theoretical and empirical basis for clinical efforts to mitigate paternal PTSD risk, though further controlled studies are needed to confirm causal efficacy.

## 1 Introduction

Preterm birth (gestational age <37 weeks) is an important issue in global perinatal health. The global incidence of preterm infants is 10.6%, and nearly 1.5 million preterm infants are born each year in China, and with the implementation of the three-child policy in China, the incidence of preterm birth is anticipated to rise. These infants, often born at the threshold of viability, exert profound psychological impacts on their parents and the entire family, leading to considerable stress and challenges (1–3). Molloy et al. (4) stated that the survival of very low birth weight preterm infants (<1,500 g) is highly dependent on rescue care in the neonatal intensive care unit (NICU). The establishment of the NICU, including the noise of the medical instrumentation, the uncertainty of the newborn’s vital signs, the physical isolation from the infant, and the high cost of medical care, significantly improves the preterm infants’ survival rates, but the unexpected event of preterm labor often causes great stress and challenges for parents (5, 6).

The mental health of fathers of preterm infants, as one of the core family members, has long been neglected in clinical research and practice. Traditionally, mothers are considered to be the main psychological victims of preterm birth, but recent studies have found that fathers’ anxiety levels during NICU stay are not significantly different from those of mothers, but their emotional expression tends to be more internalized, manifested by overwork, avoidance of communication, or somatization symptoms (e.g., insomnia and headache) (7). This “burden of silence” may lead to an imbalance in family support functions and even exacerbate the risk of maternal and infant depression, and the incidence of post-traumatic stress disorder (PTSD) in fathers is as high as 20%–35%, or even comparable to that in mothers, but this phenomenon is still under-recognized (8, 9). Theoretical perspectives suggest that perceived social support can be a critical buffer against trauma-related distress and anxiety in various health crisis contexts, highlighting its potential protective role for fathers in the NICU. As one of the psychological sequelae of preterm birth, its core symptoms include intrusive memories (e.g., recurrent flashbacks of resuscitation scenes), persistent avoidance (e.g., refusal to visit the infant), negative cognitive and emotional changes (e.g., self-blame or affective numbing), and hypervigilance (e.g., heightened sensitivity to the sound of medical sirens) (10). McKeown et al. (11) stated that fathers assume the role of “pillar of strength” in crisis, resulting in emotional distress and reduced help-seeking behaviors, and that the NICU system of care often focuses on the mother and the baby, and fathers are often marginalized as “bystanders” or “financial providers,” with limited opportunities to participate in parenting, exacerbating feelings of powerlessness and helplessness. Pace et al. (12) showed that fathers’ PTSD was significantly and negatively associated with mother–infant attachment quality, marital satisfaction, and children’s long-term behavioral problems, suggesting that neglecting fathers’ mental health may have a knock-on negative effect on the family ecosystem. Therefore, actively analyzing the relevant factors affecting its occurrence and taking intervention measures are significant for the harmony of couple relationship and family stability. Kangaroo care, also known as skin-to-skin contact, refers to the care of preterm infants by their mothers or fathers in a manner similar to that of marsupials such as kangaroos, and as an economic and effective nursing measure, it can reduce the mortality rate of preterm infants and promote their neurological development (13). However, the clinical analysis of factors related to the occurrence of PTSD in fathers of preterm infants born in the NICU has rarely been reported, and the mechanism of kangaroo care on fathers’ mental health has not been clarified. Based on this, this study intends to quantitatively assess the factors related to the occurrence of PTSD in fathers of preterm infants born in the NICU and test the intervention effect of kangaroo care by using a mixed research methodology, aiming to optimize the intervention strategy, reduce the long-term psychosocial risk of preterm families, fill the research gaps on the role of fathers in the family support system, and provide a new perspective for the construction of the family cycle’s health support network.

## 2 Materials and methods

### 2.1 Ethics statement

This study was approved by the Institutional Review Board and Ethics Committee of Xiamen Xianyue Hospital. For the retrospective component (Phase 1), written informed consent was waived by the ethics committee as only anonymized and de-identified data were extracted from existing medical records, and the study posed no foreseeable risk to participants. For the prospective intervention component (Phase 2), written informed consent was obtained from all participating fathers prior to their enrollment. Participants were fully informed about the study aims, procedures, potential risks and benefits, and their right to withdraw at any time without affecting their or their infant’s medical care. This procedure complies with the Declaration of Helsinki and local ethical regulations for biomedical research involving human participants.

### 2.2 Research design

This study employed a two-phase design. Phase 1 was a retrospective cross-sectional analysis of 251 fathers of preterm infants admitted to the NICU of our hospital between July 2023 and July 2024 as study subjects. Based on the annual admission records of our NICU, this sample represents approximately 78.4% of all eligible fathers during the study period. The data of the study subjects were obtained from the medical record system, and they were divided into two groups according to the presence or absence of PTSD [assessed by the Chinese version of the Perinatal Posttraumatic Stress Disorder Questionnaire (PPQ-C) scores]: the group without occurrence (*n* = 189) and the group with occurrence (*n* = 62). Phase 2 was a prospective, quasi-experimental pre–post intervention study without a concurrent control group. The design was selected based on feasibility and clinical constraints in the NICU setting. We acknowledge that the absence of a control group limits causal inference, and improvements may be influenced by factors such as the natural course of psychological adjustment over time (time effects), spontaneous changes in infant clinical status (e.g., weaning from respiratory support and resolution of acute medical issues), increased attention from staff during the intervention (Hawthorne effect), and the potential for repeated assessment to influence responses (testing effects). These limitations are explicitly discussed in the manuscript. All fathers who were eligible and consented to participate in Phase 2 received the kangaroo care intervention. Assessments were conducted at baseline (T1: within 48 h of enrollment, prior to any kangaroo care session) and after the completion of the intervention period (T2: within 72 h following the final kangaroo care session, approximately 2–3 weeks after T1 depending on the infant’s clinical stability and the scheduling of the five to eight kangaroo care sessions).

This study was not preregistered in a clinical trials registry, as it was initiated prior to the implementation of mandatory registration policies for observational and quasi-experimental studies at our institution. However, the study protocol was reviewed and approved by the institutional ethics committee prior to data collection. All procedures were conducted in accordance with the approved protocol, and no deviations from the original design occurred during the implementation of the study.

The observational component (Phase 1) is reported following the STROBE (Strengthening the Reporting of Observational Studies in Epidemiology) guidelines where applicable for cross-sectional studies. The intervention component (Phase 2) is reported in line with key elements of the CONSORT extension for non-randomized trials (CONSORT-NPT), acknowledging the absence of a concurrent control group.

### 2.3 Inclusion criteria

Father-related criteria: (1) Age ≥18 years; (2) conscious, cognitively clear, and possessing basic literacy and communication skills; and (3) willing to participate and able to complete the scale assessments. Infant-related criteria: (4) Gestational age <37 weeks; (5) admitted to the NICU for ≥5 days after birth; and (6) clinically stable, defined as no requirement for mechanical ventilation, vasopressor support, or surgical intervention within 48 h prior to enrollment; hemodynamically stable (heart rate and blood pressure within normal ranges for postmenstrual age); and no evidence of active seizures, sepsis, or necrotizing enterocolitis. Data completeness criterion: (7) Complete clinical data available, including demographic information, infant medical records, and scale assessment results.

Exclusion criteria: Father-related exclusions: (1) History of major chronic diseases (e.g., malignant hypertension and cancer) or sensory impairments (visual or auditory) and (2) experienced other traumatic events (e.g., accident and loss of a family member) within the past 6 months. Infant-related exclusions: (3) Infant death during the study period and (4) diagnosis of major congenital anomalies (e.g., congenital heart disease, neural tube defects, and chromosomal abnormalities).

### 2.4 Data collection

Relevant data were collected through the registration records of the medical record system, including the birth weight of the child [normal body mass (2,500–4,000 g)/abnormal body mass] [extremely/ultra-low birth mass (<1,500 g), low birth mass (1,500–2,499 g), and large birth mass (>4,000 g)], the sex of the child (male/female), the gestational age at birth of the child, method of oxygen administration to the child (no oxygen/others), whether or not the child is malformed (yes/no), method of delivery (natural delivery/cesarean section), age, occupation (unemployed/others), education level (high school and below/college and above), family income (≤3,000 yuan/>3,000 yuan), childcare experience (yes/no), marital status (married/other), payment method (self-payment/other), family residence (rural/urban), and whether or not the pregnancy was planned (yes/no).

### 2.5 Sense of disease uncertainty

Illness uncertainty was assessed using the Chinese version of the Parental Perception of Uncertainty Scale (PPUS). This version has been previously validated in Chinese parental populations, demonstrating strong construct validity and cultural appropriateness (14). The scale comprises 31 items across four subscales: ambiguity, complexity, lack of information, and unpredictability. Each item is rated on a five-point Likert scale, with total scores ranging from 31 to 155; higher scores indicate greater illness uncertainty. In the present sample, the scale demonstrated good internal consistency, with a Cronbach’s α coefficient of 0.89.

### 2.6 Social support

Social support was measured using the Social Support Rating Scale (SSRS), a widely used instrument in Chinese populations with established validity and reliability (15). The scale includes 10 items assessing objective support, subjective support, and support utilization. Total scores range from 12 to 66, with higher scores indicating greater perceived social support. In this study, the SSRS showed acceptable internal consistency (Cronbach’s α = 0.82).

### 2.7 Depressed mood

Paternal depressive symptoms were assessed using the Edinburgh Postnatal Depression Scale (EPDS). Although originally developed for mothers, the EPDS has been culturally adapted and validated for use in Chinese fathers during the perinatal period (16). In the current sample, the EPDS demonstrated good internal consistency, with a Cronbach’s α coefficient of 0.87. The scale consists of 10 items, each rated on a four-point scale (0–3) based on symptom severity over the past week, yielding a total score ranging from 0 to 30. Higher scores indicate greater severity of depressive symptoms. Consistent with prior research in fathers and to avoid diagnostic presumption, we refer to the construct measured as depressive symptoms.

### 2.8 PTSD assessment criteria

Screen-positive PTSD symptoms were measured using the PPQ-C (17). This instrument is based on the diagnostic criteria of the *Diagnostic and Statistical Manual of Mental Disorders (4th edition)* (*DSM-4*) (18) and consists of 14 items corresponding to the intrusive memories, avoidance, and hypervigilance dimensions. Each item is scored from 0 to 3, with a total score ≥ 19 indicating likely PTSD (referred to as screen-positive in this study). The PPQ-C has been previously validated in Chinese perinatal populations, demonstrating good content and construct validity (17). In the present sample, the scale also showed good internal consistency, with a Cronbach’s α coefficient of 0.85, further supporting its reliability in this specific context.

### 2.9 Kangaroo nursing measures

Prerequisites for kangaroo care: Infant criteria: (1) Stable vital signs (heart rate, respiration, and temperature within normal ranges); (2) for non-oxygen-dependent infants: oxygen saturation >88%; (3) for oxygen-dependent infants: oxygen saturation ≥85% in a calm state; and (4) no active infection or unstable medical condition. Father criteria: (5) No upper respiratory tract infection or contagious illness, and (6) willing and able to participate in the intervention. Environmental preparation: A dedicated kangaroo care space was set up within the NICU, ensuring privacy, an ambient temperature of 22–24 °C, a relative humidity of 55%–65%, and equipped with a reclining chair, soft pillows, warm blankets, footstools, and gentle background music to promote relaxation.

Training and education: Nursing staff received standardized training on kangaroo care through lectures and self-study modules, covering its origins, evidence base, benefits, and procedural steps. Fathers were educated one-on-one by trained nurses using posters, verbal explanations, and practice with an infant mannequin prior to the first session.

Implementation procedure: The father wore a front-open shirt and sat in a recliner at a 40°–60° angle. The infant, dressed only in a diaper and cap, was placed in an upright or semi-reclined position on the father’s bare chest, with a light blanket covering the back. The father supported the infant’s buttocks with one hand and placed the other hand on the infant’s back. During the session, which lasted approximately 1 h (one sleep cycle), the father was encouraged to talk, touch, and maintain eye contact with the infant. The infant remained connected to a multiparameter monitor for continuous heart rate and oxygen saturation monitoring. A trained nurse supervised the session and intervened immediately if hypoxemia, apnea, or other adverse events occurred.

Fathers were encouraged to participate in kangaroo care sessions daily, contingent upon infant stability and father availability. Each session lasted approximately 60 min. The total intervention period extended until the infant was medically stable enough for transfer to a step-down unit or discharge planning had commenced, typically resulting in five to eight sessions per father over a 2- to 3-week period. Post-intervention assessments (T2) were conducted within 72 h after the final session.

### 2.10 Observation indicators

(1) For Phase 1 (cross-sectional analysis): Collect the occurrence of PTSD in the fathers of preterm infants in the NICU, compare the relevant information of the occurrence group and the non-occurrence group, carry out univariate and multivariate logistic regression analysis of the indicators of difference, construct a nomogram to analyze the predictive value of each factor, and carry out internal validation via receiver operating characteristic (ROC) curves. (2) For Phase 2 (intervention study): Compare the positive rate of PTSD symptoms, disease uncertainty scores, social support scores, and depressive symptom scores of the study subjects before (baseline) and after the implementation of the kangaroo care intervention, and analyze the associated changes.

### 2.11 Statistical processing

In this paper, data were calculated using SPSS 25.0 statistical software. For Phase 1 (cross-sectional analysis): Count data were expressed as *n* (%) and compared using the χ² test or Fisher’s exact test as appropriate. Normally distributed continuous data were expressed as mean ± standard deviation (x̄ ± s) and compared using independent samples *t*-tests; non-normally distributed continuous data were expressed as median (interquartile range) [M (P25, P75)] and compared using the Mann–Whitney *U* test. *p* < 0.05 was considered statistically significant. A univariate analysis of factors was performed, followed by a multivariate logistic regression analysis, based on which a nomogram (column-line graph) model was constructed, and ROC curves were plotted for internal validation to verify the model’s discrimination. Calibration was assessed using the Hosmer–Lemeshow goodness-of-fit (GOF) test.

For Phase 2 (intervention study, pre–post comparison): Paired *t*-tests were used for normally distributed continuous data (e.g., disease uncertainty and social support), the Wilcoxon signed-rank test was used for non-normally distributed continuous data (e.g., depressive symptoms), and McNemar’s test was used for the categorical PTSD symptom positivity rate. To assess the magnitude of change, effect sizes were calculated: Cohen’s *d* was used for parametric comparisons (interpretation: small ≥0.2, medium ≥0.5, large ≥0.8), and the matched-pairs rank-biserial correlation (*r*) was used for non-parametric comparisons.

For Phase 2, given the lack of a control group, we conducted additional sensitivity analyses to examine potential confounding by infant clinical progress. We compared pre–post changes in fathers whose infants showed significant clinical improvement (e.g., weaned off oxygen and weight gain) versus those whose infants remained medically complex. No statistically significant interaction was found (*p* > 0.05), suggesting that infant health changes during the study period did not substantially account for the observed paternal psychological improvements. However, this analysis cannot fully exclude the influence of unmeasured confounders such as time effects, staff attention, or other concurrent psychosocial interventions.

### 2.12 Psychometric considerations

The selection of established, psychometrically validated instruments in this study (PPUS, SSRS, EPDS, and PPQ-C) aligns with best practices in behavioral health research. This approach is consistent with the methodological rigor employed in the development and evaluation of context-specific scales grounded in theoretical models, ensuring robust measurement of key constructs under investigation.

## 3 Results

### 3.1 Unifactorial analysis affecting the occurrence of PTSD in fathers of preterm infants born in the NICU

Comparison of the screen-positive group and screen-negative group regarding illness uncertainty, social support, depressive symptoms, child’s birth weight, occupation, and educational level revealed statistically significant differences (χ²/*t*/*Z* = 6.317, 5.383, 5.279, 7.364, 8.808, and 8.358, *p* < 0.05), but the differences in the comparison of the remaining indicators were not statistically significant (*p* > 0.05), as shown in **Table 1**.

**Table 1:** Single factor analysis affecting the occurrence of PTSD in fathers of premature infants in NICU.

Index		Genetic set (*n* = 62)	Non-occurrence group (*n* = 189)	χ²*/t/Z*	*p*
Disease uncertainty mark (points)		79.95 ± 20.88	63.11 ± 17.26	6.317	<0.001
Social support mark (points)		34.75 ± 7.97	40.11 ± 6.38	5.383	<0.001
Depressed mood mark (points)		10.00 (8.00, 13.00)	8.00 (6.00, 10.00)	5.279	<0.001
Child birth weight (*n*)	Normal body mass	12	72	7.364	0.007
	Abnormal body mass	50	117		
Gender of child (*n*)	Male	28	90	0.113	0.737
	Female	34	99		
Gestational age (weeks)		30.75 ± 3.11	30.89 ± 3.15	0.305	0.761
Oxygen administration for the child (*n*)	Anaerobic	6	16	0.086	0.8770
	Other	56	173		
Whether the child is malformed (*n*)	Yes	1	1	0.694	0.405
	No	61	188		
Delivery mode (*n*)	Spontaneous delivery	19	82	3.152	0.076
	Cesarean section	43	107		
Age (years)		29.38 ± 3.11	29.01 ± 3.47	0.747	0.456
Occupation (*n*)	Unemployed	16	20	8.808	0.003
	Other	46	169		
Educational level (*n*)	High school and below	41	85	8.358	0.004
	College or above	21	104		
Household income (*n*)	≤3,000 yuan	14	29	1.722	0.189
	>3,000 yuan	48	160		
Parenting experience (*n*)	With	9	47	2.886	0.089
	Without	53	142		
Marital status (*n*)	Married	54	169	0.254	0.614
	Other	8	20		
Payment method (*n*)	Self-financing	15	30	2.197	0.138
	Other	47	159		
Family place of residence (*n*)	Village	26	69	0.585	0.445
	City	36	120		
Whether to plan pregnancy (*n*)	Yes	45	152	1.701	0.192
	No	17	37		

### 3.2 Multifactorial logistic regression analysis affecting the occurrence of PTSD in fathers of preterm infants delivered in the NICU

As a result of the logistic regression analysis, it was found that disease uncertainty, social support, depressed mood, child’s birth weight, occupation, and literacy level were independent risk factors affecting the occurrence of PTSD in fathers of preterm infants delivered in the NICU [odds ratio (OR) = 1.053, 0.893, 1.300, 2.564, 2.939, and 2.389, 95% confidence interval (CI) = 1.033–1.073, 0.853–0.935, 1.175–1.437, 1.280–5.138, 1.411–6.122, and 1.312–4.348, *p* < 0.05], as shown in **Table 2**.

**Table 2:** Multivariate logistic regression analysis of influence on the occurrence of PTSD in NICU preterm fathers.

Influencing factor	*B* value	BSE	BWald χ²	*BP*	BOR value	OR value 95% CI
Disease uncertainty mark	0.052	0.010	28.726	<0.001	1.053	1.033–1.073
Social support mark	−0.113	0.023	23.385	<0.001	0.893	0.853–0.935
Depressed mood mark	0.262	0.051	26.168	<0.001	1.300	1.175–1.437
Child birth weight	0.942	0.355	7.050	0.008	2.564	1.280–5.138
occupation	1.078	0.374	8.293	0.004	2.939	1.411–6.122
Educational level	0.871	0.306	8.119	0.004	2.389	1.312–4.348

### 3.3 Risk prediction model construction and validation

**Figure 1:**
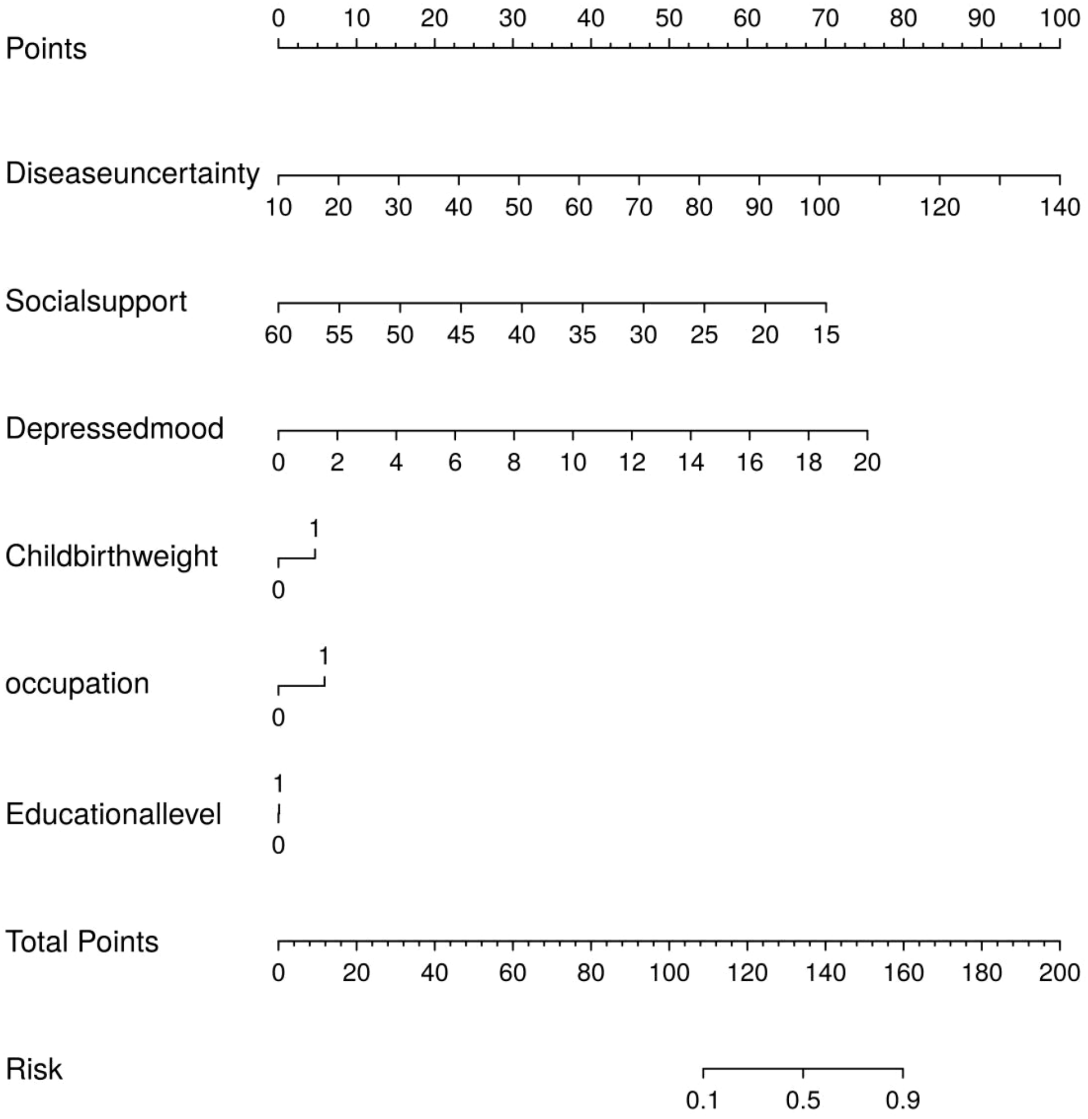
Construction of the nomogram risk prediction model.

**Figure 2:**
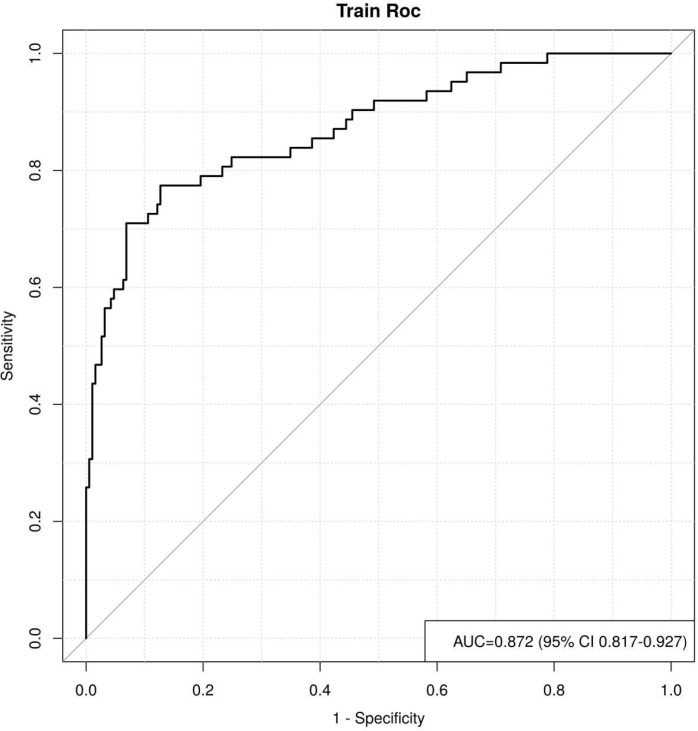
ROC curve.

Based on multifactorial logistic regression analysis, a nomogram was constructed using six independent risk factors (disease uncertainty, social support, depressed mood, child birth weight, occupation, and literacy level) to predict the probability of PTSD in fathers of preterm infants. In this visual model (**Figure 1**), each variable is assigned points; the sum of these points corresponds to a scale at the bottom, allowing for individualized risk assessment. The Hosmer–Lemeshow GOF test was used to assess the logistic regression model fit, the ROC curve was used for internal validation, and the results showed that the area under the curve (AUC) value was 0.872, with a 95% CI of 0.817–0.927, which indicated that the predictive model had a high overall accuracy (see **Figure 2**).

### 3.4 Comparison of indicators before and after the implementation of kangaroo care disease uncertainty, social support, and depressed mood

**Figure 3:**
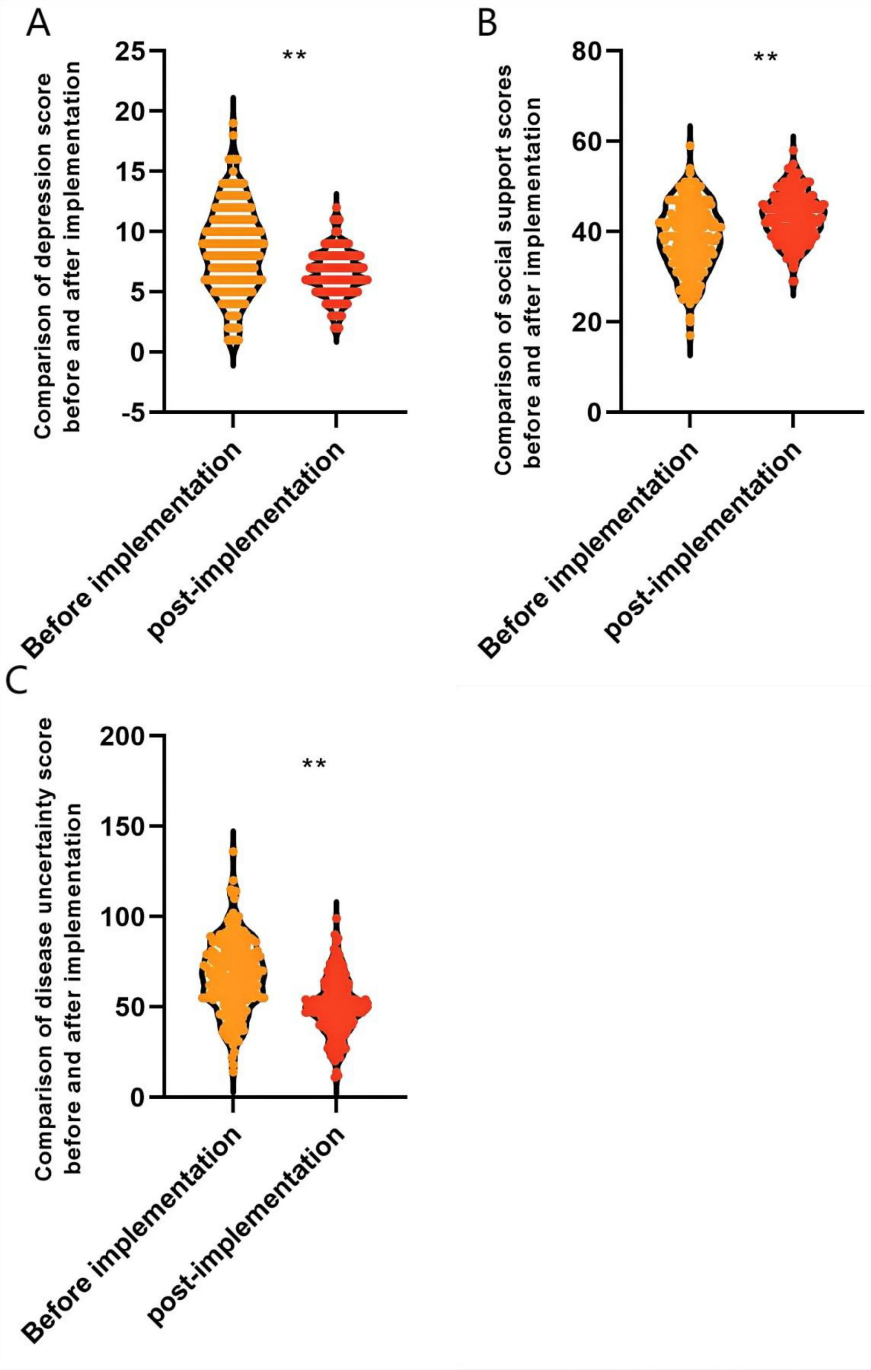
Comparison of indexes before and after kangaroo nursing. **(A)** Comparison of depression score beforeand after implementation; **(B)** Comparison of social supportscores before and after implementation; **(C)** Comparison of disease uncertainty score before and after implementation. **p < 0.001.

After the implementation of the kangaroo care intervention (assessed within 72 h post-intervention, approximately 2–3 weeks after baseline), the father’s screen-positive PTSD symptom rate [7.97% (20/251) vs. 24.70% (62/251)], the score of disease uncertainty (67.27 ± 19.07 vs. 50.12 ± 15.11) points, the social support score (38.78 ± 7.17 vs. 43.25 ± 5.16) points, and the depressive symptom score (8.54 ± 2.9 vs. 6.48 ± 1.88) points were compared with the pre-intervention period, and the difference was statistically significant (paired *t*-test for disease uncertainty and social support; Wilcoxon signed-rank test for depressive symptoms; McNemar’s test for PTSD positivity rate; all *p* < 0.05), thus indicating that the implementation of kangaroo care was associated with improvements in the study subjects’ PTSD, uncertainty about the illness, depressive symptoms, and social support. Effect size analyses indicated large magnitudes of change: disease uncertainty (Cohen’s *d* = 0.99), social support (Cohen’s *d* = -0.72; negative sign indicates increase in score), and depressive symptoms (matched-pairs rank-biserial *r* = 0.58). These changes are shown in **Table 3**, **Figure 3**.

**Table 3:** Comparison of indexes before and after kangaroo nursing (*n*, %).

Group	*Bn*	PTSD symptom positive rate
Before implementation	251	62 (24.70)
Post-implementation	251	20 (7.97)
*t*	–	25.712
*p*	–	<0.001

## 4 Discussion

The prevalence of postpartum depression in fathers of newborns is 7.4%, and it is higher in fathers of preterm infants, with a prevalence of postpartum depression of 56.86%, and the prevalence of PTSD in fathers of infants hospitalized in the NICU is 47% (19, 20). Sellouti et al. (21) defined PTSD as a mental disorder with delayed onset after severe traumatic stress, marked by intrusive re‑experiencing, persistent avoidance, negative cognitive and mood changes, and heightened arousal and reactivity. PTSD-positive screening in fathers of preterm infants not only affects infants’ clinical outcomes and parent‑child bonding, but also impairs fathers’ physical and mental health and increases family psychological burden. Thus, identifying influencing factors and implementing targeted measures is key to preventing and managing clinically significant PTSD symptoms in this population. Therefore, the key to preventing and treating clinically significant PTSD symptoms is to actively analyze the factors affecting screen-positive PTSD symptoms in fathers and take corresponding measures.

The data of this study show that the difference between the occurrence group and the non-occurrence group in terms of disease uncertainty, social support, depression, child birth weight, occupation, and education is statistically significant (*p* < 0.05); the results of logistic regression analysis show that the above indexes are independent risk factors affecting the occurrence of PTSD in fathers of preterm infants born in the NICU (*p* < 0.05). Previous studies have shown that parents with lower education cannot avoid the influence of emotional factors on their own behavior better, are not rational enough to face events such as preterm birth and low quality of pregnancy, have a relatively lower awareness of internet use and online consultation to learn relevant knowledge, and lack knowledge about the development and regression of the condition of preterm infants, resulting in a higher risk for the occurrence of PTSD, which further supports the thesis of this study (22). Preterm infants have underdeveloped oral muscles and uncoordinated sucking and swallowing functions, and it often takes a long time to achieve independent oral feeding, which leads to prolonged hospitalization of preterm infants, implying higher hospitalization costs and increased financial pressure on the family. Malouf et al. (23) stated that the income status of unemployed fathers is more unstable compared to that of employees and self-employed individuals, among others, and a stable income is an important guarantee for preterm infants’ hospitalization and subsequent rehabilitation, and thus the risk of PTSD is higher. Stable income is an important guarantee for the hospitalization and subsequent rehabilitation of preterm infants; thus, the incidence of PTSD is relatively higher in fathers of preterm infants who are unemployed. Preterm infants have immature organ systems and often suffer from respiratory and feeding disorders. Poor birth status is associated with more complications, lower survival, poorer external adaptation, and more difficult treatment. Repeated anxiety over their infant's survival and health exposes fathers to severe psychological stress, which may lead to PTSD. These results are consistent with those reported by Malin et al. (24) arguments. According to the study by Salomè et al. (25), mothers of preterm infants typically focus on postpartum recovery after delivery, while fathers often assume primary responsibility for understanding their infant's condition and providing informed consent for medical treatments. When treatment progresses poorly or unexpectedly, fathers frequently develop emotional disturbances, including insomnia, impaired behavior, irritability, anxiety, and other depressive symptoms. This emotional state not only affects the father’s mental health, but may also exacerbate his sensitivity and the intensity of his reaction to traumatic events, thereby increasing the risk of developing PTSD. Illness uncertainty refers to the confusion and uneasiness that individuals experience when confronted with an illness due to a lack of adequate information or understanding (26). The admission of a child to the NICU will produce a strong stimulus for the father, and when a cognitive framework cannot be established for the relevant disease and hospitalization events, it will be difficult to make an accurate prediction of the outcome, leading to dysfunction in the role of the father of the child, and the inability of the father to cope up with immense psychological stress and show a sense of uncertainty about the disease, which may in turn trigger the father’s fear, interfere with his normal cognitive functioning and coping strategies, and increase the likelihood of PTSD. Previous studies have indicated that social support usually comes from friends, faith groups, other social relationships, or family members, and that those fathers who receive emotional, physical, and psychological support show better behavioral status and coping abilities than those who do not (27). Social support among fathers of neonates primarily derives from family resources, especially spousal parenting confidence and competence. Mothers with strong parenting abilities provide substantial emotional support to fathers, alleviating excessive worry about post-discharge care challenges for preterm infants and thereby reducing paternal psychological stress. Fathers who perceive higher social support often maintain more stable family relationships, which enhances their parenting self-efficacy and coping capacity. This allows them to accept the treatment trajectory and future health outcomes of preterm infants with greater confidence. Conversely, low social support increases feelings of isolation and helplessness, rendering fathers more vulnerable and powerless when facing traumatic events and elevating their risk of PTSD. These observations are supported by findings from Haeusslein et al. (28). In this study, we constructed a column-line graphical prediction model by combining various factors and transforming complex multifactorial regression equations into graphs, resulting in the visualization and readability of abstract data endings, evaluating the approximate probability of the occurrence of poor prognosis by means of different predictor variables, and plotting ROC curves for internal validation, which showed that the AUC value was 0.872, with a 95% CI of 0.817–0.927, showing that the difference between the predicted probability and the actual probability was not statistically significant, indicating that the predictive model can provide substantial clinical support, thus suggesting not only that the occurrence of PTSD in fathers of preterm infants in the NICU is influenced by multidimensional factors but also that healthcare professionals should develop intervention strategies at multiple levels after preterm infants are admitted to the NICU for treatment as their condition permits, thus achieving the prevention and treatment goals. Beyond its procedural aspects, kangaroo care can be conceptualized as a theory-driven behavioral intervention that may enhance paternal self-efficacy and promote adaptive health behaviors. Drawing from behavioral models such as Pender’s Health Promotion Model, interventions that actively engage individuals in health-promoting activities (like kangaroo care) can strengthen perceived benefits, self-efficacy, and interpersonal support, thereby fostering positive psychological outcomes. Kangaroo care is a scientifically supported, cost-effective, and humanized model of care that originated from observations of kangaroo nurturing behavior. Initially implemented in Colombia in settings with limited medical resources, it has since been widely recognized and promoted by the World Health Organization (29, 30). The data of this study show that following the kangaroo care intervention, the rate of positive PTSD symptoms, disease uncertainty score, and depressive symptom score of fathers were lower than at baseline, but the social support score was higher than at baseline (*p* < 0.05), suggesting a potential beneficial association, which is in line with the arguments of Saltzmann et al. (31). This may be explained by the fact that kangaroo care provides fathers with increased opportunities to observe and interact with preterm infants. Through close contact, fathers gradually gain a clearer understanding of their infant's clinical condition and witness improvements over time. Perceiving the infant's needs also promotes emotional and behavioral engagement in fathers. Meanwhile, consistent parent -child interaction facilitates positive role adaptation among these fathers and encourages them to actively seek support from nursing staff. With continuous accompaniment and guidance, fathers acquire more parenting-related knowledge and skills, which in turn enhances their perceived social support. The improvement of preterm infants’ depression may be related to the skin-to-skin contact between fathers and preterm infants during the implementation of kangaroo care. Murthy et al. (32) noted that when fathers of preterm infants enter the NICU, they can directly observe the infant’s treatment environment and understand the infant's behavioral and physiological characteristics. Through parent -child interaction, early parent -child attachment is established, which improves paternal confidence and competence in adapting to their parental role. Fathers also develop a better ability to recognize and respond appropriately to their infant’s potential needs. This process helps reduce depressive symptoms in these fathers and ultimately improves their psychological and behavioral functioning. Through the above data in this study, it can be seen that disease uncertainty, social support, and depressive symptoms are all independent risk factors for screen-positive PTSD symptoms in fathers of preterm infants born in the NICU. Thus, kangaroo care measures were associated with a reduction in the rate of screen-positive PTSD symptoms, potentially by influencing the above indicators. However, the absence of a control group in this quasi-experimental design precludes definitive causal attribution of these improvements solely to the kangaroo care intervention. Several potential confounding factors must be considered when interpreting these findings. First, the natural trajectory of psychological adaptation (time effects) may lead to spontaneous improvement in paternal distress as the infant’s condition stabilizes and the family adjusts to the NICU environment. Second, improvements in infant clinical status (e.g., weaning from respiratory support and resolution of acute medical issues) during the intervention period could independently reduce paternal anxiety and distress. Third, the increased attention and support from nursing staff during kangaroo care sessions (Hawthorne effect) may have contributed to the observed improvements, regardless of the specific intervention content. Fourth, repeated exposure to the assessment instruments (testing effects) may have influenced fathers’ responses over time. While sensitivity analyses suggested that infant clinical improvement alone did not fully explain the findings, these other confounding factors cannot be entirely ruled out. Future studies with randomized controlled designs and active control conditions are needed to establish causal efficacy. However, it is worth noting that kangaroo care in China requires the collaboration of many aspects, including the likelihood of its implementation to the family, the family’s subjective willingness/comprehension/health, and the guidance of professionals. During the initial implementation of monitoring equipment, healthcare providers should offer hands_on guidance regarding operational skills. Throughout the procedure, staff should acknowledge and encourage fathers’ efforts, while also actively prompting and supporting them to participate appropriately in care.

Although the above clinical values were achieved, there are still shortcomings: (1) the study mostly relies on scale assessment, which may be influenced by the subjective report of the father and lacks objective physiological indicators; furthermore, while validated instruments were used, some (e.g., EPDS) were not originally designed and validated specifically for fathers in the NICU setting; (2) the intervention component (Phase 2) lacked a control group, which limits the ability to establish causality and attribute observed changes definitively to the kangaroo care intervention itself; (3) only the fathers of preterm infants were selected as the subjects of this study, and the mothers of preterm infants may be investigated at the same time at a later stage, so as to analyze the correlations and differences between the parents; and (4) although the nomogram model has been widely used in medicine and other fields, the prediction accuracy is completely dependent on the reliability of its underlying statistical model. If the statistical model is defective or not applicable to specific scenarios, then the prediction results of the nomogram model may also be affected. Crucially, the nomogram was developed and internally validated using data from a single center, which limits its generalizability to other NICUs with different patient populations, clinical practices, or cultural contexts. Therefore, while the model demonstrated good discrimination in our sample, external validation in independent, multicenter cohorts is essential before it can be recommended for widespread clinical use. (5) Future research would benefit from developing and validating father-specific psychometric tools tailored to the unique stressors and experiences within the NICU context, drawing on established methodological frameworks used in other health behavior domains.

## 5 Conclusion

In summary, multiple factors, including illness uncertainty, social support, depressive symptoms, infant birth weight, paternal occupation, and education level, influence the risk of screening positive for PTSD symptoms in fathers of NICU preterm infants. The constructed nomogram provides a practical tool for early risk stratification. The findings from the quasi-experimental intervention suggest that kangaroo care is a promising and feasible intervention associated with positive changes in paternal screen-positive PTSD symptoms, illness uncertainty, depressive symptoms, and perceived social support. However, because of the lack of a control group and the potential influence of confounding factors such as time effects, infant clinical improvement, and staff attention, these results should be interpreted with caution and do not imply definitive causal efficacy. Future rigorously controlled, multi-center trials are warranted to establish causal effectiveness, clarify mechanisms, and optimize implementation strategies for kangaroo care in supporting paternal mental health. Additionally, external validation of the proposed nomogram in diverse, multi-center populations is essential to confirm its generalizability and clinical utility across different NICU settings and populations.

## Data Availability

The original contributions presented in the study are included in the article/supplementary material. Further inquiries can be directed to the corresponding author.
